# Whole body vibration therapy and cognitive functions: a systematic review

**DOI:** 10.3934/Neuroscience.2023010

**Published:** 2023-05-18

**Authors:** Nisha Shantakumari, Musaab Ahmed

**Affiliations:** 1 College of Medicine, Ajman University, Ajman, United Arab Emirates; 2 Center of Medical and Bio-allied Health Sciences Research, Ajman University, Ajman, United Arab Emirates

**Keywords:** whole body vibration, vibration, cognition, neurocognition, cognitive tests

## Abstract

Whole Body Vibration has been found to induce physiological changes in human subjects, improving their neuromuscular, respiratory and cardiovascular functions. Evidence from animal research prove that whole-body vibration appears to induce changes in molecular and cellular levels to alter cognitive functions in mice. There is evolving evidence for a potential value of whole body vibration in improving cognition and preventing the development of age-related cognitive disorders in humans. However, literature on the biological consequences of whole-body vibration on the human brain is scanty. If so, gathering the available evidences would help decide the possibility of designing appropriate whole-body vibration protocols to extend its application to induce neurocognitive enhancement and optimize its effects. Therefore, a systematic review of the literature was performed, consulting the ProQuest, MEDLINE and Scopus bibliographic databases, to summarize the available scientific evidence on the effects of whole-body vibration on cognitive functions in adults. Results of the review suggest that whole-body vibration therapy enhances a wide spectrum of cognitive functions in adults although there isn't enough evidence available yet to be able to design a standardized protocol to achieve optimum cognitive enhancement.

## 1. Introduction

The human body has its own vibratory frequency ranging between 5 to 20 Hz. Exposure of human body to vibrations in the range of 20–70 Hz may be considered as mild stressful stimuli initiating adaptive effects in different body systems [1]. This principle has been the basis on which the concept of whole-body vibration (WBV) therapy has evolved over the past two decades. WBV has now come to be used as an alternative or supplement to conventional physical training. Essentially, the WBV therapy platforms deliver repeated, rapid and short intermittent oscillations to the body at variable frequencies (commonly ~15–60Hz) causing vertical, horizonal or alternating displacements which are transmitted through the legs or other body parts. The vibrations could be delivered while the patient is standing or sitting on a chair place on the vibrating platform.

There is solid evidence to prove that subjecting the body to these challenging frequencies of vibration through customized whole-body vibration results in beneficial effects on cardiovascular and musculoskeletal systems similar to those observed after physical training. Recent evidence suggests that WBV exercise could be an alternative exercise modality to improve body composition, muscle strength, postural stability, bone mass, sensorimotor performance and cardiovascular function in various populations [2]–[4].

Studies in both humans and mice have demonstrated that physical exercise induces structural and functional changes in the brain and has enormous effect on cognition [5],[6]. Exercise especially improves executive functions like planning, working memory, attention, problem solving, inhibition, multi-tasking and mental flexibility [7]. The positive effects of WBV on cognition were explored primarily in animal models. Histological and morphometric analyses of mice brain revealed that WBV induces neurogenesis, neuronal plasticity and alteration in transmission across neurotransmitter systems, all of which improve cognitive performance [8]–[10]. The data on the effect of WBV on cognition in humans is minimal and inconclusive. The purpose of this review is to (1) summarize the available data on the effects of WBV on cognitive function in humans and (2) gather evidence on its potential use as an alternative strategy to exercise in enhancing cognition.

## 2. Methods

Following the recommendations proposed by PRISMA, a systematic review was performed to assess the effect of whole-body vibration therapy on cognitive capacity in adult humans.

### 2.1. Study selection

#### 2.1.1. Inclusion criteria

Study design: Experimental Clinical trials

Language: English

Population: Adults (18 years or older) with or without mental or neurological disorders

Intervention: Whole body vibration therapy using vibrating platform

Outcomes: Cognitive effects that are attributable to whole body vibration are reported

#### 2.1.2. Exclusion criteria

Study design: Reviews, commentary papers, dissertations, letters to the editor, case reports, book, protocols, news, opinions, notes, conference abstracts, repeated studies

Full article not available or not in English language

Population: Animal studies; populations under 18 years of age

Intervention: Vibration related to occupation or using vehicles

Outcomes: Studies where outcomes relating specifically to effect on cognition are not reported

### 2.2. Search terms and databases

The review sought to explore the effect that whole-body vibration has on cognition. The term vibration has been used in conjunction with not only whole-body vibration using vibration plates but also vibrations applied to limbs alone. Hence the need to use the term” whole body vibration” to narrow down the search options to intentional whole-body vibration exposure. Based on this the two scientific keywords were identified with (MeSH) terms as: “whole-body-vibration” and “cognition” interspersed by the Boolean Operator ‘AND’. A comprehensive literature search was performed for eligible studies by searching the following electronic databases: ProQuest, MEDLINE and Scopus. Articles published up to December 2022 were searched.

### 2.3. Data extraction

**Figure 1.**
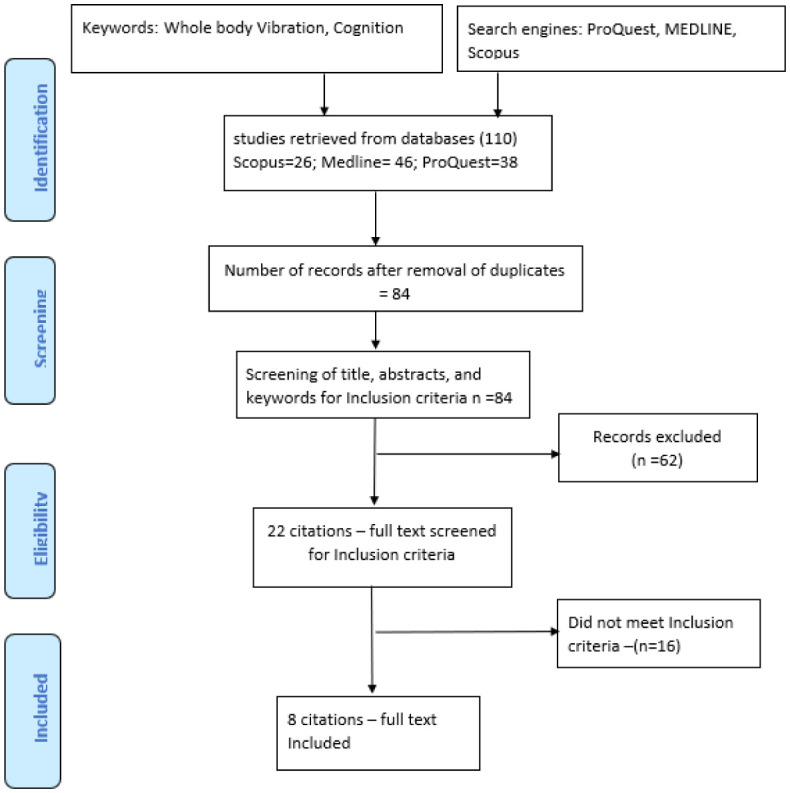
Flowchart showing overview of the review process.

The results of searches by the two independent review authors were combined for each database to form a single dataset. Duplicates within and between datasets were removed. The title and abstracts were screened according to the eligibility criteria for relevance. The results of this initial screening by both authors were cross-referenced between the two review authors and full-text records obtained for all potentially relevant reports. A total of 110 articles were retrieved from the electronic databases. After removing 26 duplicates, the screening of titles, abstract and keywords of 84 articles led to exclusion of 62 papers. The full texts of the remaining 22 articles were screened and the 8 articles found to meet the inclusion criteria were included in the analysis. The main exclusion criteria were animal studies, occupational or automobile vibration and non-availability of full text. The overview of the review process is represented in Figure 1.

## 3. Observations from the review

A total of eight studies were included in this review. All the studies stated similar objectives evaluating the effects of whole-body vibration on neurocognitive functions. Five of the studies included in this review were conducted in healthy adults [11]–[15]. Table 1 summarizes the profile of participants of the studies included in the review. The study population in the remaining three studies had morbidities like lumbar hyper lordosis, ADHD and senile dementia [16]–[18]. The study population in majority of studies were in the age group of 18–42 years [12],[14]–[17]. While Hugo rosado and Ki-Hong Kim targeted elderly population (age > 75 years), Boerema included population in a wide age range of 42–99 years.

**Table 1.** Profile of participants.

**Study**	**Type of study**	**Number of participants**	**Mean age(yrs.)**	**Gender**	**Condition of the participants (healthy/comorbid conditions)**
**Regterschot GR, et al., 2014**	Single group repeated-measures experimental design	133	20.5±2.2	21M and 112F	Healthy
**Boerema AS et al., 2018**	Randomized controlled trials	Experimental group: 18, Control group: 16	Experimental group: 65.8(42-99), control group 66.0 (45-90)	15M, 19F	Healthy
**Fuermaier AB, et al., 2014**	Nonrandomized repeated-measures experimental design	Experimental group: 17, Control group: 83	Experimental group: 24.2 ±1.9 (21 to 28); Control group: 22.5±3.7 (8 to 31)	Experimental group: 8M, 9 F; Control group: 40M, 43F	Experimental group: Attention deficit hyperactivity disorder Control group: Healthy
**Fereydounnia S et al., 2017**	Single group repeated-measures experimental design	Experimental group: 15, Control group: 15	Experimental group: 28.93 ± 3.45 Control group: 28.07 ± 2.68	30F	Experimental group: Lumbar hyper lordosis Control group: Healthy
**Rosado, H et al., 2021**	Randomized controlled trials	Experimental group 1: 18, Experimental group 2: 19, Control group: 19	75.4 ± 5.6	47F, 9M	Healthy
**Paddan GS, 2012**	Repeated-measures Latin-square experimental design	20	M: 29.0 ±10.2 F:33.8 ± 8.5	10M, 10F	Healthy
**Amonette WE, 2015**	Randomized crossover study	12	Mean age: 28.2± 6.4 years;	8M, 4F	Healthy
**Ki-Hong Kim, 2018**	Randomized controlled trials	Experimental group: 9, Control group: 9	Experimental group: 79.22±4.02; Control group: 81.44±3.75	18F	Senile dementia

M, Males; F, Females

### 3.1. Vibration protocol

The effects of WBV are highly variable and are dependent strongly on parameters characterizing the mechanical vibrations. It is imperative to ensure that the doses of vibration delivered are optimal to induce positive physical and biological responses. Table 2 summarizes the vibration protocol adopted by the studies reviewed.

The dose is quantified in terms of volume and intensity measured by the duration of vibration exposure, its amplitude and frequency [19],[20]. All studies included in the review used low amplitude of vibration (<5 mm) that has been reported to be widely employed in sports training protocols [21].

None of the studies reviewed used high frequency vibrations that have been proven to be deleterious to human body. Regterschot et al., Fuermaier et al., Fereydounnia et al., Amonette et al., and Boerema et al. used a frequency of 30 Hz in their study, which according to available literature is the best in enhancing performance [12],[13],[15]–[17],[21]. Paddan et al., and Rosado et al., used lower frequencies [11],[14]. While no beneficial effect was recorded by Paddan et al., Rosado et al. found significant improvement in reaction time and dual task performance time. The beneficial effects of low frequency vibration in Rosado's study could possibility be attributed to fact that vibration was delivered not in isolation but alongside with psychomotor intervention program and over longer period of time i.e 24 weeks.

**Table 2.** Whole body vibration protocol.

**Reference**	**Frequency of Sessions × Duration of Program**	**No. of Vibration Bouts × Duration per Bout**	**Rest periods**	**Frequency (Hz)and Amplitude (mm) of Vibration Signals**	**Type of vibration and device type**	**Posture adopted**	**Protocol for Control Group/Session**
**Regterschot GR, et al., 2014**	Single session	6 bouts × 2min	Rest periods of 120s after bout of vibration and control session	Frequency: 30 Hz, Amplitude: 0.5 mm	Vertical vibration: Vibe 300 from Tonic Vibe, Nantes, France.	Seated on a chair mounted on vibration platform	Same group with no vibrations during control sessions
**Boerema AS et al., 2018**	4 sessions/week, for 5 weeks.	1 bout x 4 min	None	Frequency: 30 Hz, Amplitude: 0.5-1 mm	Vertical vibration: Pactive Motion (type Rolstoelpod)	Seated on chair mounted on vibration platform	4 sessions/week, for 5 weeks. Frequency: 1 Hz, Amplitude: 0,5-1mm
**Fuermaier AB, et al., 2014**	Single session	4 bouts × 2min	4 interspersed rest periods of 2 min each and 7 breaks of 3 min each between each rest and vibration session.	Frequency: 30 Hz Amplitude: 4 mm	Vertical vibration: Vibe300, Tonic Vibe, Nantes, France.	Seated on chair mounted on the vibration platform	Single session Frequency: 30 Hz, Amplitude: 4mm
**Fereydounnia S et al., 2017**	Single session	5 bouts × 1min	5 rest periods of 30s each	Frequency: 30 Hz Amplitude: 5 mm	Vertical vibration: Power Plate vibration platform (Power Plate USA)	Standing	Single session Frequency: 30 Hz Amplitude: 5 mm
**Rosado, H et al., 2021**	3 times /week for 24 weeks	Vibration of 3 min/session and ending with 6 min/session,	Resting time between series 60 s.	Frequency (Hz) increased from 12.6 to 15, Amplitude: 3 mm.	Side-alternating vibration: Galileo® Med35	Standing with bent knees	No vibration
**Paddan GS, 2012**	Once a day for 5 days	Each session 18 min including exposure to 3 different frequencies at any given 5 seat angles	None	3 test stimuli: Frequency: 2–8 Hz; 8–14 Hz and 14–20 Hz, Amplitude: Not given	Vertical vibration: Servo test man-rated	Seated at one of the at 5 different seat back angles of 90°, 67°, 45°, 22° and 0°.	No vibration
**Amonette WE, 2015**	Two sessions of different vibration type seperated by not more than 2 weeks	5 sets of any one type of vibration x 2min	None	Frequency: 30 Hz Amplitude: 4 mm for both vertical and horizontal vibration	Vertical vibration: Power Plate vibration platform (Power Plate USA) Rotational sessions: Galileo 2000 vibration platform	Static hip-width stance squats, with the knees positioned at a 45 degrees angle of flexion.	No vibration
**Ki-Hong Kim, 2018**	5 sessions /week for 8 weeks.	5 sets x 2min	60 s between sets	Began with frequency of 20 Hz, and increased gradually by 5 Hz every 2 weeks.	Vertical vibration: Whole body vibration exerciser (VM-10, Korea)	Standing squatting and sumo squat position	No vibration
				Amplitude: Not given			

Hertz (Hz); Whole body vibration (WBV)

### 3.2. Effect of posture

The intensity of vibrations transmitted to the head varies widely with the posture adopted during exposure to whole body vibration. It was found that squatting at 110-degree knee angle results in the least transmissibility of vibrations to the head when compared to other relatively high or low squat positions [22]. Amonette et al., recorded that WBV caused no improvement in cognitive functions of subjects adopting a posture of static hip-width stance squats, with the knees positioned at a 45 degrees angle of flexion [15].

Naser Nawayseh found that while standing on a vibrating platform, a bent knee posture results in a weaker transmission of vibrations and standing with one foot to the front and other to the back transmits the vibrations more efficiently to the head [23]. The participants in the studies conducted by Fereydounnia et al. and Rosado et al. were made to stand on a vibrating platform during the session. At the end of the intervention they were found to have significant improvement in reaction time, anticipatory skills, mobility, and dual-task performance. The combination of WBV and psychomotor intervention program was shown to have a medium to large size effect on reaction time, mobility, and dual-task [11],[16].

The transmission of vibrations was found to be greater in persons seated when compared to those who were standing [24]. Regterschot et al., Boerema et al. and Fuermaier et al. had their participants seated on a chair during the intervention. Administration of vibration to these seated subjects resulted in significant improvement in color -word interference test and stroop difference test [11],[12]. Paddan et al. on the contrary recorded a vibration induced decline in cognitive function of seated individuals. They varied the angle of the seat back rest and found the psychomotor performance was adversely affected in all participants across all backrest angles with the greatest decline at backrest angle of 22.5 degrees [14].

These conclusions raise a possibility that the higher intensity of vibrations transmitted to the head in seated posture could be potentially reducing the benefits or even causing deleterious effects on cognition.

### 3.3. Effect of morbidities

Ki-Hong Kim studied the effects of WBV on women with senile dementia and recorded a significant improvement in their Mini-Mental State Examination scores after 8 weeks of intervention. This improvement has been attributed to an increased blood flow in the cerebral circulation. These effects correspond to the observation that regular physical exercise enhances cognitive functions and prevents the progressive cognitive decline in patients with dementia [18].

Fuermaier et al. compared the effect of WBVT on cognitive functions of 17 adults having ADHD with 83 healthy adults. Just two minutes of WBV treatment resulted in an acute improvement in attention in patients with ADHD with a medium effect size as opposed to the small effect size noted in healthy controls. Attention problems being the most common cognitive deficits in persons with ADHD, this observation is of significance from a clinical perspective [17].

Fereydounnia et al. noted that individuals with lumbar hyper lordosis when subjected to WBV showed no significant improvement in reaction time and anticipatory skills except for their Visual complex choice reaction time. The authors attributed this to the fact that improvement in anticipatory skills only happens over a period of time with the formation of new synapses. They also state that some of the baseline cognitive measures were already good and hence significant improvement is not anticipated post intervention [16].

Differences in the spinal curvature, and muscle tension may be partially responsible for the differences in the transmission of vibration across vertebral column. Flexion of the hip and decreased lumbar lordosis increases the transmission of vibration through the spine [27]. Hence another plausible explanation could have been that disorders of spine could dampen the transmission of vibrations to the cranial region and could hence reduce its beneficial effect in improving cognition.

### 3.4. Tests of cognition and measure of effect

Table 3 summarizes the cognitive tests employed, aspects of cognition measured and the observations after intervention. A wide range of tests have been developed to assess cognitive functions and quantify the degree of cognitive impairments. Few frequently used cognitive function tests include stroop test for selective attention, and inhibition; Digits Forward and Backward subtests for working memory and Trail Making Test for mental flexibility [28]. Regterschot et al., Boerema et al. and Fuermaier et al. recorded the cognitive benefits of WBVT using one or more of these three tests in their study population [12],[13],[17]. Fereydounnia et al. and Rosado et al. used reaction time demonstrate the effect of WBV on attention, execution and psychomotor speed [11],[16]. All the above-mentioned tests are also commonly used to assess the effect of aerobic training interventions on attention, processing speed, executive function, working memory and psychomotor speed [29]. Using similar neurocognitive tests could help in drawing some comparisons between the effect of these two interventions among similar study groups.

ImpAct test used by Amonette et al., provides composite scores as a measure of verbal memory, visual memory, motor processing speed, and reaction time [15]. Paddan's et al. study employs the NASA- Task Load Index (NASA-TLX) questionnaire that measures cognitive workload and relies on self-recall of cognitive workload after completion of the task [14]. There isn't enough data to supports its validity on measure cognitive function. It is suggested that this increased cognitive workload may serve as a predictor of cognitive decline [30] Hence the instrument can be considered an indirect measure of cognition.

All except one of the selected studies tested the cognitive functions immediately after the intervention protocol. There were no reports on changes in the cognitive effects of vibration over time after the cessation of the intervention. We would however need data supporting longer lasting effects of cognitive enhancement to establish it as a treatment approach. Rosado repeated the tests of cognition after an interval of 12 weeks post intervention and found that though there are short term benefits, the effect is not retained and wears off over a period of time. This suggests the need for implanting a long-term protocol to sustain the cognitive benefits of the intervention.

**Table 3.** Measure of Cognitive function.

**Reference**	**Tests used**	**Outcomes measured**	**Observation after intervention**
**Regterschot GR, et al., 2014**	1. Stroop test (color-block test, color -word interference test, stroop difference test) 2. Digital span backward test	1. Pyschomotor speed, selective attention and inhibition 2. verbal short memory/working memory	No difference in results of color block test and Digital span backward test between WBV and control sessions. Performance on color -word interference test and stroop difference test improved after WBV.
**Boerema AS et al., 2018**	1. Stroop test (word test, color-block test, color -word test) 2. Digital Memory span forward and backward 3. Trail making test (part A and B)	1.selective attention and inhibition 2. verbal short memory 3. cognitive flexibility	Improvement in all tests except Trial making A test. Statistically significant improvement noted only in Stroop color word test.
**Fuermaier AB, et al., 2014**	Stroop Color-Word Interference task (Color Block Test and the Color-Word Interference Test)	Selective attention and inhibition.	Significantly improved attention performance with small size (d = 0.44) in controls and a medium effect (d = 0.64) in individuals with ADHD.
**Fereydounnia S et al., 2017**	1. Visual choice reaction time test 2. Visual complex choice reaction time test 3. Auditory choice reaction time test 4. Anticipatory skill with high and low speed	Reaction time and anticipatory skills	Improvement in all test parameters in both groups. Significant improvements in control group the auditory complex choice reaction time and anticipatory skill with high speed after vibration. In the lumbar hyper lordosis group, significant difference only in visual complex choice reaction time
**Rosado, H et al., 2021**	Reaction time (simple and choice reaction), Cognitive timed up and go	Reaction time (simple and choice reaction), execution and psychomotor speed	Significant improvements in improvements in reaction time and dual-task performance (medium to large effect size). The interventions' effects were no longer evident after the 12-week no-intervention follow-up period
**Paddan GS, 2012**	NASA-TLX subscales: Mental workload	Mental workload	22.5° seat backrest angle adversely affect the psychomotor performance during vibration. 2–8 Hz, had a detrimental effect on both overall performance tracking and reaction times for the cognitive tasks
**Amonette WE, 2015**	Computerized test battery (ImPACT)	Computerized test battery (ImPACT): verbal memory, visual memory, motor processing speed, and reaction time	No effect on visual or verbal memory, reaction time, or impulse control measured using ImPACT. Motor processing speed increased
**Ki-Hong Kim, 2018**	K-MMSE, EEG	Orientation about time and space, memory, concentration and calculation, linguistic skills, understanding and judgment. Brain activation on EEG	Significant improvement in all cognitive functions and EEG activation

Attention deficit hyperactivity disorder (ADHD); NASA task load index (NASA-TLX); Immediate Post concussion Assessment and Cognitive Test (ImPACT); Korean-Mini-Mental Status Examination (K-MMSE), Electroencephalography (EEG)

## 4. Strengths and limitations of the study

The quality of the research articles that have been evaluated is one of the strong points of this review. The literature search and selection of the studies were done by two independent reviewers, which increases the power of the study and reduces errors. This review has some limitations. Firstly, the literature search was limited to the three major electronic databases: ProQuest, MEDLINE and Scopus, no other databases were searched. Therefore, additional relevant studies might have been missed. Secondly, free search engines were used for literature search and hence the inability to fully retrieve some articles. Thirdly, we only included articles in English.

## 5. Conclusions

There is limited data on neurocognitive effects of whole-body vibration. Studies compared in our manuscript differed significantly in terms of vibration protocols (intensity and duration), comorbidities of study population, posture adopted and timing of cognitive tests. This poses difficulties in comparing the evidence analyzed and arriving at any standardized protocols that could be suggested for this intervention. Regardless of the limitations in comparison, it can safely be inferred that whole-body vibration, has a high potential for positive interference in improving cognitive abilities both in healthy individuals and in those with cognitive comorbidities.
